# Japanese version of the Brief Sense of Community Scale: construct validity and reliability

**DOI:** 10.1186/s40359-022-01017-x

**Published:** 2022-12-12

**Authors:** Kyung-Ran Yu, Keigo Asai, Taku Hiraizumi, Koubun Wakashima

**Affiliations:** 1 Faculty of Education, Miyagi Gakuin Women’s University, Sendai, Japan; 2 Graduate School of Education, Hokkaido University of Education, 1-15-55, Shiroyama, Kushiro-shi, Hokkaido 085-8580 Japan; 3 School of Nursing, Miyagi University, Sendai, Miyagi Japan; 4 Graduate School of Education, Tohoku University, Sendai, Japan

**Keywords:** Brief Sense of Community Scale (BSCS), Confirmatory factor analysis, Gender, Japanese, Psychological sense of community, Reliability, Validity

## Abstract

**Background:**

The Brief Sense of Community Scale is a widely accepted eight-item scale that measures the four dimensions of sense of community; however, the factor structure of the Japanese version of this scale has never been confirmed. In this study, we demonstrated the reliability and validity of the Japanese version of the Brief Sense of Community Scale.

**Methods:**

After completing the back translation of the scale, a sample of 993 Japanese individuals completed the Japanese version of the Brief Sense of Community Scale.

**Results:**

The results indicate that the Japanese version of the Brief Sense of Community Scale is comparable to the original scale and demonstrated adequate goodness-of-fit for both the four-factor and second-order models, which demonstrates its validity. Nonetheless, it remains necessary to consider possible cross-national cultural concerns when utilizing the scale.

**Conclusions:**

The Japanese version of the Brief Sense of Community Scale will contribute toward the creation of a community in which all members feel comfortable.

## Background

Sense of Community (SOC)—sometimes also called “psychological sense of community”— refers to “a feeling that members have of belonging, a feeling that members matter to one another and to the group, and a shared faith that members’ needs will be met through their commitment to be together” [1]. The concept of SOC has mainly evolved from the theoretical framework of McMillan and Chavis [1], which emphasizes the following four core dimensions of SOC: need fulfilment (a sense that members’ needs will be met by the resources received by their group membership); membership (a shared sense of personal relatedness or belonging to the community); influence (a sense of mattering to group members or community, such as by making a difference in the group); and emotional connection (a belief among members related to a shared history, place, time, and experiences).

Nonetheless, some researchers have argued that the four dimensions of SOC are not relevant for the measurement of SOC in a statistical sense [2, 3]; it has frequently been reported that measurements including reversal items or response methods cannot provide statistical clarity of the SOC for any samples [2, 4]. Additionally, the necessity of a measure including fewer items, which can be utilized in various community contexts, has been raised [2, 5].

To address these problems, Peterson et al. [5] created the Brief Sense of Community Scale (BSCS) and confirmed its reliability and validity. Comprising only eight items, the BSCS can not only measure the four SOC dimensions proposed by McMillan and Chavis [1] but can also represent a robust factor structure [5]. Recently, BSCS has gained attention for its applicability in various community studies, such as those among youth [6, 7], LGBT individuals [8], and residents [5]; moreover, it has been translated to several non-English languages [9]. BSCS is widely used in Asia, including China [10–13], Korea [14], Vietnam [15], and Japan [16], as well as in the Western community. However, problems exist in the BSCS used in Asia, such as the translation process being unclear [10–19] and back translate being performed but not examined for validity [20]. In addition, the factor structure of the BSCS has not been examined, and many studies use the BSCS as a one-factor [10–20]. We believe that it is necessary to clarify the factor structure of the BSCS in Asia and to verify its validity to make cross-national comparisons with the Western community. We conducted the present study in Japan as the first step, and the findings can be compared with those in other regions of Asia in the future.

It is noteworthy that the most SOC studies in Japan developed their own measures instead of adopting Western ones [21–23]. This is because the strong influence of Japan’s unique socio-cultural contexts, such as “*ie*” (household) and “*mura*” (village community), can hinder the accurate translation of a measure to Japanese [21]. Nonetheless, the independent measures developed in Japan cannot be considered suitable for application in future cross-national studies on SOC. The Japanese version of the BSCS will resolve this issue of the generalization of SOC measurements.

Considering this situation, we developed a Japanese version of the BSCS and examined its reliability and validity within a sample of Japanese community residents. To verify the “criterion-related validity,” we also used the Sense of Community Scale 2 (SCI-2), Community Consciousness Scale (CCS [21, 22]), and Center for Epidemiologic Studies Depression Scale (CES-D [24]). Below, we briefly explain why each of the three scales were used for the criterion-related validity test.

(i) SCI-2: As a McMillan-Chavis four-factor structure was confirmed in SCI-2 by increasing the number of items, eliminating inverted items, and making changes to the required responses, it is expected that all factors of SCI-2 would be positively correlated with those of the Japanese version of the BSCS.
(ii) CCS: CCS is a widely accepted measure of SOC that was developed in Japan and includes the roles of the government and initiatives by citizens of the community. [21, 22]
(iii) CES-D: SOC has been shown to improve an individual’s mental health [1, 25, 26], and CES-D, a measure of depression, is negatively correlated with BSCS. [5, 11, 12, 18]

## Methods

### Participants

The survey was outsourced to an online survey agency (Cross Marketing Co., Ltd.). Participants were Japanese residents from various age groups and regions who provided their consent to participate. The survey was conducted such that the number of men and women and those in each age group were approximately equal. In total, 1000 Japanese people were recruited to participate in the survey in November 2021. Excluding a few suspicious responses (such as those with the same answers throughout the questionnaires), the responses of 993 participants (498 men and 495 women) were used for further statistical analysis. The mean and standard deviation of the ages were 49.55 years and 16.74 years, respectively. Among the participants. 33.53% were aged 18–39, 31.13% were aged 40–59, and 33.33% were aged 60 or older. In terms of educational level, 2.11% were less than high school educated, 29.41% had completed high school, 19.64% had graduated from junior college or vocational school, 43.20% had earned a bachelor’s degree, and 5.64% had a master’s degree or higher. A total of 53.07% of the participants lived in the eastern region of Japan (Hokkaido, Tohoku, Kanto region) and 49.63% in the western region (Chubu, Kinki, Chugoku, Shikoku, Kyushu, Okinawa region). In addition, the eastern and western regions have different cultures (e.g., food flavoring) [27]. Thus, both regions are geographically and culturally different. As the Chubu region can be considered either to the east or the west [28], it was classified as the western region in this study.

## Measures

### Japanese version of the BSCS

The BSCS, created by Peterson et al. [5], comprises eight items scored on a 5-point Likert-type scale (ranging from 1 = strongly disagree to 5 = strongly agree) to assess the four dimensions of SOC (needs fulfilment, membership, influence, and emotional connection). The scale’s reliability was confirmed by an internal consistency of Cronbach’s *α* = 0.85 ~ 0.92 [5, 7]. We translated the BSCS into Japanese after obtaining permission and performed the standard procedure of back-translation with advice from Dr. Peterson.

### Sense of Community Index 2 (SCI-2)

SCI-2 is a 24-item self-report scale that adopts the theoretical framework of McMillan and Chavis [1]; its four subscales are “reinforcement of needs,” “membership,” “influence,” and “shared emotional connection.” [29] The scale’s reliability was confirmed by an internal consistency of Cronbach’s *α* = 0.91 for the entire scale and 0.79 ~ 0.86 for the subscales [30].The SCI-2 items are scored on a 4-point Likert scale ranging from 0 (not at all) to 3 (completely), with six items under each subscale.

### Short version of Community Conscious Scale

The short version of community conscious scale is a 12-item self-report questionnaire [22]. This scale is a short version of the CCS [21, 31] considers SOC to be a multifaceted concept that included the roles of government and citizen agency in the community. The items are scored on a 5-point Likert-type scale ranging from 1 (disagree) to 5 (agree). This scale comprises the following four subscales, each including three items: solidarity (e.g., “I want to participate in voluntary activities in my community”), self-determination (e.g., “In the problem solving in the community, it is important to build up an equal partnership between the residents and administration”), attachment (e.g., “Because I live temporarily in this community, I have neither the concern of nor the attachment to my community”), and dependency on others (e.g., “It is acceptable that the residents leave the activities in improving their living environment in the community to more dedicated people”).

### Japanese version of the Center for Epidemiologic Studies Depression (CES-D)

The Japanese version of the CES-D is a 20-item self-report scale that measures depression under the following four subscales: “somatic complaints,” “depressed affect,” “positive affect,” and “interpersonal problems” [32]. Participants respond to the 20 items based on their experiences within the past week on a 4-point Likert scale ranging from 0 (rarely or none of the time, less than 1 day) to 3 (most or all the time, 5–7 days).

## Procedures

This study was approved by the Ethics Committee of the authors’ university. Participation was entirely voluntary. Before administering the questionnaire, the participants were informed of the survey overview and terms of confidentiality, and their consent was obtained. Cross Marketing awarded the participants with reward points for completing the questionnaire.

## Statistical analyses

Confirmatory factor analysis (CFA) was performed using the Mplus 8.1 statistical software package [33]. Additionally, the maximum likelihood estimator (ML) was applied for CFA estimation in this study. To test the goodness-of-fit, we conducted comparative fit index (CFI), Tucker–Lewis index (TLI), root mean square error of approximation (RMSEA), standardized root mean square residual (SRMR), Akaike’s information criterion (AIC), Bayesian information criterion (BIC), and Akaike’s Bayesian information criterion (ABIC). The cut-off values for acceptable model fit used in this study are: RMSEA ≧ 0.10 for poor fit and < 0.06 for good fit; CFI > 0.90 for acceptable fit and > 0.95 for good fit; and SRMR < 0.10 for acceptable fit and < 0.08 for good fit [34, 35]. For additional analyses, multiple-group CFA was conducted to examine gender and region of residence (eastern or western region) invariance. Furthermore, measurement invariance was tested by creating three models: a configural model (no constraints), metric invariance model (with item loading constraints to be equal across groups), and scalar invariance model (with item loadings and item intercepts with simultaneous constraints to be equal across groups). Following the hierarchy of these nested models, they were compared with each other. We focused on the ratio of χ^2^ to its degree of freedom, ΔCFI, ΔRMSEA and ΔSRMR as indicators in the model comparisons. Model invariance is indicated by Δχ^2^/Δ*df* of less than or equal to 5 and ΔCFI smaller than 0.01, accompanied by ΔRMSEA smaller than 0.015 and ΔSRMR smaller than 0.03 [36–38]. For the comparison of factor means, we followed guidelines [39] and demonstrated Δ*M* and *p*-value.

Other analyses were conducted using the program R version 3.6.3 [40]. Cronbach’s alpha coefficients, McDonald’s omega coefficients, and correlations between the Japanese BSCS and other measures were established by calculating the Pearson’s correlation coefficients. All statistical analyses used two-tailed tests. For all statistical evaluations, *p* values less than 0.05 were considered indicative of significant differences.

## Results

### Factor structure

**Fig. 1:**
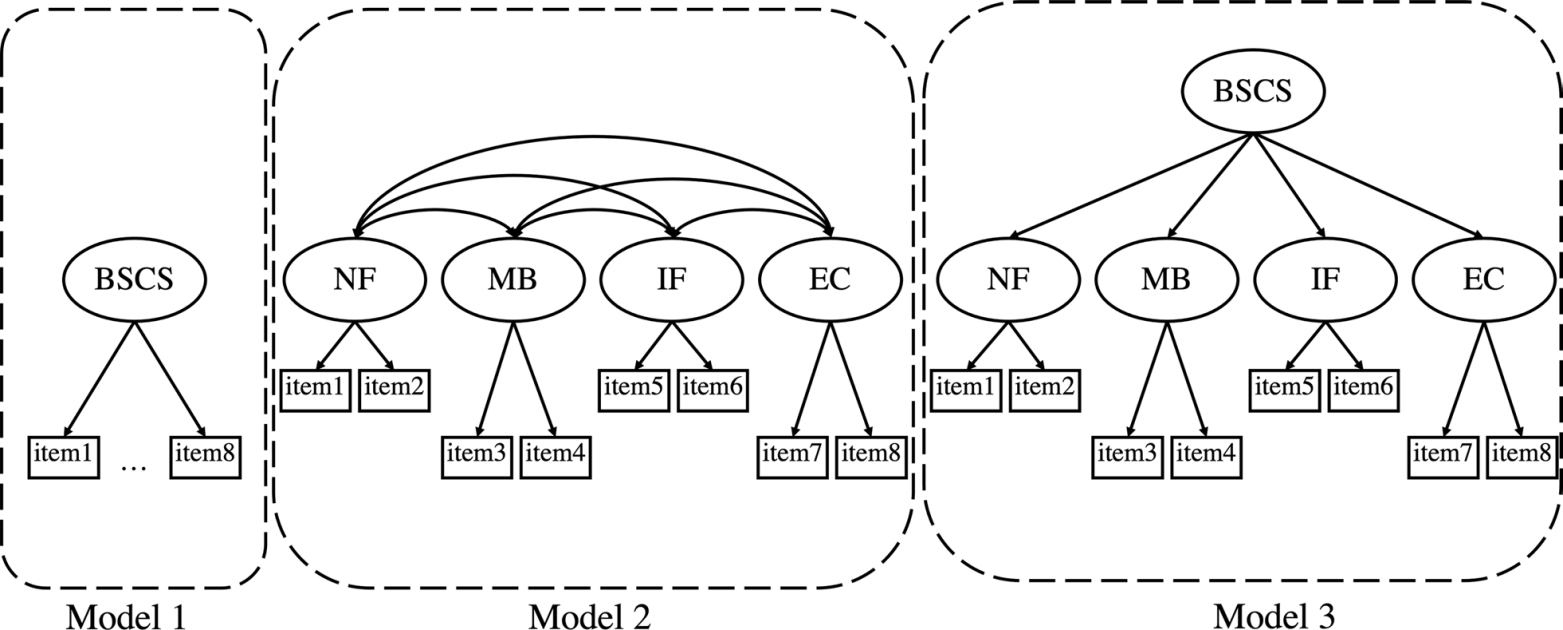
Three models in Brief sense of community scale (BSCS)

The mean scores for each item of the Japanese BSCS are shown in Table 1. The mean values for each item ranged 2.33–3.10, with standard deviations ranging 0.91–1.04. Skewness and kurtosis were smaller than 1. Confirmatory factor analyses were used to examine the goodness-of-fit. Based on the original scale validation study [5], three models were tested for use among Japanese participants (Fig. 1). As shown in Table 2, the four-factor and second-order models demonstrated acceptable fits, whereas the one-factor case did not. Therefore, although the second-order model had higher AIC, BIC, and ABIC than the four-factor model, we will examine the gender and region of residence invariances for both.

**Table 1:** Descriptive Statistics of the Japanese BSCS Items

	Mean	(SD)	Skewness	Kurtosis	Item 1	Item 2	Item 3	Item 4	Item 5	Item 6	Item 7	Item 8	NF	MB	IF	EC
Item 1	3.10	(.96)	− 0.41	− 0.12	–											
Item 2	3.06	(.94)	− 0.29	− 0.13	.80**	–										
Item 3	2.93	(1.03)	− 0.22	− 0.47	.56**	.61**	–									
Item 4	3.08	(1.04)	− 0.37	− 0.42	.53**	.57**	.86**	–								
Item 5	2.33	(1.04)	0.32	− 0.65	.27**	.29**	.41**	.37**	–							
Item 6	2.65	(.91)	− 0.12	− 0.05	.41**	.46**	.51**	.48**	.61**	–						
Item 7	2.80	(1.04)	− 0.16	− 0.64	.49**	.54**	.71**	.70**	.52**	.66**	–					
Item 8	2.86	(1.04)	− 0.18	− 0.54	.45**	.52**	.71**	.69**	.49**	.61**	.84**	–				
NF	6.16	(1.81)	− 0.38	0.00	.95**	.95**	.62**	.58**	.30**	.46**	.54**	.51**	–			
MB	6.02	(2.00)	− 0.33	− 0.34	.56**	.61**	.96**	.96**	.40**	.51**	.73**	.73**	.62**	–		
IF	4.97	(1.75)	0.19	− 0.11	.37**	.41**	.51**	.47**	.91**	.88**	.66**	.61**	.41**	.51**	–	
EC	5.66	(2.00)	− 0.21	− 0.52	.49**	.56**	.74**	.73**	.53**	.67**	.96**	.96**	.55**	.76**	.66**	–
BSCS	22.81	(6.30)	− 0.31	0.10	.71**	.76**	.86**	.83**	.63**	.75**	.88**	.85**	.77**	.88**	.77**	.90**

^**^p < .01

**Table 2:** Factor Loadings for the Japanese BSCS

Items (internal reliability)	Standardized loadings			
	1 factor	4 factor	Second-order	
BSCS total (*α* = .91/ *ω* = .91)				
Needs Fulfillment (*α *= .89/ *ω* = .89)	–		.68	
1 I can get what I need in this neighborhood	.62	.85	.85	
2 This neighborhood helps me fulfil my needs	.68	.94	.94	
Membership (*α* = .92/ *ω* = .92)	–		.86	
3 I feel like a member of this neighborhood	.86	.94	.94	
4 I belong in this neighborhood	.84	.91	.91	
Influence (*α* = .75/ *ω* = .75)	–		.79	
5 I have a say about what goes on in my neighborhood	.54	.69	.69	
6 People in this neighborhood are good at influencing each other	.68	.89	.88	
Emotional Connection (*α* = .91/ *ω* = .91)	–		.96	
7 I feel connected to this neighborhood	.88	.93	.93	
8 I have a good bond with others in this neighborhood	.86	.90	.90	

χ^2^	1312.56***	39.53***	140.76***	
*df*	20	14	16	
CFI	.785	.996	.979	
TLI	.699	.992	.964	
RMSEA	.255	.043	.089	
90 Percent CI	.243–.267	.027–.059	.075–.102	
SRMR	.081	.013	.035	
AIC	17,874.910	16,613.880	16,711.110	
BIC	17,992.528	16,760.902	16,848.330	
ABIC	17,916.303	16,665.621	16,759.401	

^***^p < .001

Multiple-group CFA was performed to examine the gender and region of residence invariances in both models. In the four-factor model, the metric invariance and configural models showed acceptable cutoff criteria (gender: Δχ^2^/Δ*df* = 1.46, ΔCFI = − 0.001, ΔRMSEA = − 0.006 and ΔSRMR = 0.016; region: Δχ^2^/Δ*df* = 0.98, ΔCFI = 0.000, ΔRMSEA = − 0.006 and ΔSRMR = 0.011). The metric invariance and scalar invariance models also showed acceptable cutoff criteria (gender: Δχ^2^/Δ*df* = 0.75, ΔCFI = 0.000, ΔRMSEA = − 0.004 and ΔSRMR = 0.001; region: Δχ^2^/Δ*df* = 1.31, ΔCFI = -0.001, ΔRMSEA = − 0.002 and ΔSRMR = 0.003). Consequently, the scalar invariance model was supported in the four-factor model (Table 3). Meanwhile, in the second-order model, the metric invariance and configural models were acceptable (gender: Δχ^2^/Δ*df* = 4.47, ΔCFI = − 0.004, ΔRMSEA = − 0.002 and ΔSRMR = − 0.019; region: Δχ^2^/Δ*df* = 4.11, ΔCFI = − 0.004, ΔRMSEA = − 0.003 and ΔSRMR = 0.010). The metric invariance and scalar invariance models also showed acceptable cutoff criteria (gender: Δχ^2^/Δ*df* = 0.78, ΔCFI = 0.000, ΔRMSEA = − 0.009 and ΔSRMR = 0.000; region: Δχ^2^/Δ*df* = 1.32, ΔCFI = − 0.001, ΔRMSEA = − 0.007 and ΔSRMR = − 0.001). Therefore, the scalar invariance model was supported in the second-order model as well (Table 3).

**Table 3:** Measurement Invariance of Four factor model and Second-order model Among Gender Groups and Region Groups for the Japanese BSCS

		χ^2^	df	CFI	RMSEA	SRMR	Δχ^2^/Δ*df*	ΔCFI	ΔRMSEA	ΔSRMR
*Gender*										
Four factor model										
Male	41.72**		14	.991	.063	.014				
Female	43.57**		14	.990	.065	.025				
Multigroup analysis										
Configural	85.29**		28	.991	.064	.020				
Metric	96.96**		36	.990	.058	.036	1.46	− .001	− .006	.016
Scalar	102.92**		44	.990	.052	.037	.75	.000	− .004	.001
Second-order model										
Male	72.77**		16	.981	.084	.027				
Female	109.03**		16	.969	.108	.045				
Multigroup analysis										
Configural	181.80**		32	.975	.097	.037				
Metric	217.54**		40	.971	.095	.056	4.47	− .004	− .002	− .019
Scalar	223.80**		48	.971	.086	.056	.78	.000	− .009	.000
*Region*										
Four factor model										
East	27.31*		14	.996	.042	.017				
West	32.90**		14	.993	.054	.017				
Multigroup analysis										
Configural	60.21**		28	.995	.048	.017				
Metric	68.07**		36	.995	.042	.028	.98	.000	− .006	.011
Scalar	78.51**		44	.994	.040	.031	1.31	− .001	− .002	.003
Second-order model										
East	75.68**		16	.982	.084	.032				
West	96.80**		16	.970	.104	.045				
Multigroup analysis										
Configural	172.48**		32	.977	.094	.038				
Metric	205.35**		40	.973	.091	.048	4.11	− .004	− .003	.010
Scalar	215.94**		48	.972	.084	.047	1.32	− .001	− .007	− .001

*p < .05, **p < .01

Finally, the factor means were compared between genders. In the four-factor model, women had lower factor means than men for both IN and EC, which were all non-significant (NF: Δ*M* = 0.02, *p* = 0.73; MB: Δ*M* = 0.01, *p* = 0.09; IN: Δ*M* = − 0.03, *p* = 0.60; EC: Δ*M* = − 0.02, *p* = 0.72). Meanwhile, in the second-order model, women had lower factor means than men, which were not significant (Δ*M* = − 0.02, *p* = 0.83). Similarly, factor means were also compared between the regions. In the four-factor model, the western region had higher factor means than the eastern region with the exception of NF, which were not significant (NF: Δ*M* = − 0.01, *p* = 0.81; MB: Δ*M* = 0.07, *p* = 0.30; IN: Δ*M* = 0.03, *p* = 0.55; EC: Δ*M* = 0.10, *p* = 0.11). Meanwhile, in the second-order model, the western region had higher factor means than eastern region, which were not significant (Δ*M* = 0.08, *p* = 0.23).Thus, we did not find differences between the factor means of men and women, in the eastern and western regions either in the four-factor or the second-order models.

### Reliability and validity of the BSCS

The descriptive statistics of the Japanese version of the BSCS are presented in Table 1, which indicates that the intercorrelations between all domains were significant, ranging from *r* = 0.41 to 0.76. The values for the internal consistency reliability were acceptable for need fulfillment (*α* = 0.89/ *ω* = 0.89), membership (*α* = 0.92/ *ω* = 0.92), influence (*α* = 0.75/ *ω* = 0.75), and emotional connection (*α* = 0.91/ *ω* = 0.91), as well as for the total BSCS scores (*α* = 0.91/ *ω* = 0.91).

Table 4 shows the correlations between demographic variables and the four domains of the BSCS, and between BSCS and the other scales. Age was significantly associated with membership (*r* = 0.16, *p* < 0.01; 95% CI 0.06 ~ 0.27) and emotional connection (*r* = 0.14, *p* < 0.01; 95% CI 0.03 ~ 0.24). Additionally, educational level was significantly associated with needs fulfilment (*r* = . 15, *p* < 0.01; 95% CI 0.05 ~ 0.25). The correlations between all domains in the Japanese BSCS and SCI-2 were significant, ranging from *r* = 0.29 ~ 0.56, *p* < 0.01. All domains of the Japanese BSCS were significantly positively correlated with solidarity (*r* = 0.39 ~ 0.54, *p* < 0.01), self-determination (*r* = 0.32 ~ 0.40, *p* < 0.01), and attachment (*r* = 0.33 ~ 0.58, *p* < 0.01) in the CCS. Dependency on others in the CCS demonstrated a significantly negative correlation with only membership (*r* = − 0.11, *p* < 0.01). All factors of the Japanese version of the BSCS, except influence, were negatively correlated with CES-D (*r* = − 0.15 ~ − 0.21, *p* < 0.01).

**Table 4:** Correlations between the Japanese BSCS and other scales

			BSCS			
	*α*	*ω*	NF	MB	IN	EC
Gender			.00	.00	− .03	− .01
Age			.07	.16**	.05	.14**
Education			.15**	.05	.10	.07
Live (month)			.04	.22**	.08	.19**
*SCI-2*						
Reinforcement of Needs	.94	.94	.42**	.49**	.49**	.56**
Membership	.92		.31**	.39**	.49**	.48**
Influence	.92	.92	.29**	.36**	.48**	.44**
Shared Emotional Connection	.93	.93	.36**	.44**	.47**	.52**
*CCS*						
Solidarity	.91	.91	.39**	.46**	.48**	.54**
Self-determination	.90	.90	.33**	.38**	.32**	.40**
Attachment	.50	.65	.42**	.58**	.33**	.56**
Dependency on others	.86	.86	.01	− .11*	− .02	− .09
CES-D	.91	.92	− .15**	− .21**	− .04	− .21**

^**^p < .01

## Discussion

We developed the Japanese version of the BSCS and confirmed its four subscales—“needs fulfilment,” “membership,” “influence,” and “emotional connection”—through analyses of its factor. For the factor structure, acceptable goodness-of-fit was confirmed in both the four-factor and second-order models. The multiple-group CFA by gender and region of residence found identical factor structures and non-differentiable factor loadings and intercept. Additionally, the factor means were not differentiable in the four-factor and second-order models. The BSCS has represented the same factor structures across various samples, such as in midwestern United states neighborhood residents [5], youth [6, 7], and LGB individuals [8]. Therefore, the factor structure of BSCS may show demonstrate robustness even in communities within different cultures and demographics. We also note that the four-factor and second-order models in this study, in addition to the previous study cases [5–8], showed adequate goodness-of-fit.

Let us discuss the correlations between the Japanese version of the BSCS and other measures, which can indicate acceptable reliability and validity of the Japanese BSCS. First, the significantly positive correlations between each factor of the BSCS and all factors of the SCI-2 indicate that the Japanese version of the BSCS corresponds to the four dimensions of McMillan and Chavis [1]. Second, in the correlations between the BSCS and CCS, we found that “solidarity,” “self-determination,” and “attachment” in the CCS were positively correlated with all factors of the BSCS; additionally, “dependence on others” in the CCS demonstrated a negative correlation with only “group membership” in the BSCS. As “dependency on others” is a factor that indicates an indifference toward local issues and reliance on unknown others to solve them [21, 22, 31], it is possible that the feeling of being a part of the community results in a decrease in the indifference toward the community. Third, CES-D was negatively correlated with the BSCS. This indicates that a sense of community is related to individual mental health in Japan, as in previous studies [5, 25, 26].

It is also remarkable that the correlations between the Japanese version of the BSCS and the demographic variables were generally consistent with those in previous studies [5, 8], and that the Japanese version of the BSCS had a factor structure in common with cases in previous studies [5–8]. Taken together, these results suggest that the BSCS has a robust factor structure, even across different cultures.

## Limitations

This study has some limitations. We note that the relationships between the SOC and “community participation” could not be evaluated. As local activities remain restricted due to COVID-19 in Japan [41], we did not continue to measure community participation as it may mislead further analysis. We hope that future studies will analyze the community participation from the perspective of the SOC.

The second limitation is that comparisons by place of residence could only be made for the broad categories of the eastern and western regions. In this study, respondents were asked about their place of residence, but only up to prefecture. It is possible that SOC may differ between those who live in urban areas and those who live in rural areas, even in the same prefecture. In the future, it will be necessary to examine differences by size of cities of residence and type of community, such as rural or urban.

The third limitation is related to the survey methodology; we used an online survey agency to collect data from participants of various ages and living across different areas of Japan. However, nonprobability online surveys, as in this study, have been noted to be less accurate than probability telephone and online surveys [42]. Therefore, it may be necessary to conduct a probability survey, or a survey of all members of a particular local community, to examine whether a similar factor structure can be obtained.

Finally, as this study was conducted on Japanese participants, it remains unclear whether the factor structure identified in the present study is also observed in other countries, particularly in Asia. In the future, it will be necessary to clarify the factor structure of the BSCS through cross-national comparisons.

## Conclusions

Despite the abovementioned limitations, this study is the first in Asia to utilize the BSCS. The Japanese version of the BSCS was found to have good internal consistency. The positive correlations with other SOC scales and negative correlations with depression indicate that the validity of the Japanese BSCS is relatively comparable to the original BSCS. The Japanese BSCS is an 8-item scale for the assessment of SOC in Japanese communities, which will contribute to the creation of communities in which members feel a sense of “belonging.” Moreover, the Japanese version of the BSCS will enable cross-national comparative studies of SOC in the future.

## Data Availability

The datasets used and/or analyzed during the current study are available from the corresponding author on reasonable request.

## Abbreviations

SOC: Sense of community
BSCS: Brief Sense of Community Scale
SCI-2: Sense of Community Scale 2
CCS: Community Consciousness Scale
CES-D: Center for Epidemiologic Studies Depression Scale
CFA: Confirmatory factor analysis
ML: Maximum likelihood estimator
CFI: Comparative fit index
TLI: Tucker–Lewis index
RMSEA: Root mean square error of approximation
SRMR: Standardized root mean square residual
AIC: Akaike’s information criterion
BIC: Bayesian information criterion
ABIC: Akaike’s Bayesian information criterion
